# Enteroaggregative *Escherichia coli* foodborne outbreak in Shandong Province, China (2023): comprehensive epidemiology and genomic resistance profiling

**DOI:** 10.3389/fmicb.2025.1577277

**Published:** 2025-06-27

**Authors:** Lu Liu, Shuang Wang, Xiaolin Yu, Yuzhen Chen, Yanru Chen, Gaoxiang Sun, Ziqing Liu, Lixiao Cheng, Huaning Zhang, Zengqiang Kou

**Affiliations:** ^1^Infection Disease Control of Institute, Shandong Center for Disease Control and Prevention, Jinan, China; ^2^Shandong Provincial Key Laboratory of Intelligent Monitoring, Early Warning, Prevention and Control for Infectious Diseases, Jinan, China

**Keywords:** Enteroaggregative *Escherichia coli* (EAEC), foodborne outbreaks, antimicrobial resistance, whole-genome sequencing, “One Health” approach

## Abstract

**Introduction:**

Enteroaggregative *Escherichia coli* (EAEC) is an emerging and intricate diarrheagenic bacterial pathogen responsible for acute and persistent diarrhea in children, adults, and travelers. Despite its clinical significance, the global understanding of EAEC outbreaks and pathogen characteristics remains limited due to its complex epidemiological profile.

**Methods:**

This study presented a retrospective analysis of a foodborne diarrheal outbreak caused by EAEC in a county school in Shandong Province, China in 2023 by using epidemiological investigation and comprehensive genomic analysis techniques.

**Results:**

A total of 15 EAEC isolates were identified, including 13 from stool samples, one from sauced beef, and one from flies. Epidemiological and phylogenetic analyses pinpointed the EAEC isolates from sauced beef as the primary causative agent of the outbreak. Genomic comparisons revealed significant genetic consistency across nine outbreak-associated strains, particularly in virulence gene profiles, antimicrobial resistance gene profiles, molecular typing, and genetic evolution.

**Discussion:**

These findings underscored the utility of whole-genome sequencing in elucidating the genetic diversity of EAEC within specific environments and tracing its origins, thereby supporting targeted interventions such as infection control and prevention strategies. Notably, the study also identified an extensively drug-resistant (XDR) EAEC strain from flies in the canteen, harboring both the *mcr-1* and *bla_CTX-M-132_* resistance genes. This unexpected discovery highlighted the critical importance of applying the “One Health” approach, emphasizing the need for continuous surveillance of antibiotic-resistant bacteria in animals and environmental sources to mitigate potential public health risks.

## Introduction

Diarrheal diseases continue to pose a substantial global public health challenge, particularly in developing regions, where they remain a leading cause of morbidity and mortality among children (Nhambirre et al., 2025). Current research estimates that diarrheal illnesses are responsible for over 1.6 million deaths annually, with children under the age of five accounting for more than a quarter of these fatalities (Dereje et al., 2024). This burden is especially pronounced in low-income regions of sub-Saharan Africa, South Asia, and Latin America, where inadequate sanitation and significant economic constraints exacerbate the severity and fatality of these diseases (GBD 2021 Diarrhoeal Diseases Collaborators, 2024).

Among the bacterial pathogens implicated, *Escherichia coli* (*E. coli*) stands out as a prevalent cause of both acute and chronic diarrhea (Akhtar et al., 2024). While *E. coli* is a natural and typically harmless inhabitant of the intestinal microbiota in humans and warm-blooded animals, it can become pathogenic under specific conditions (GBD 2021 Diarrhoeal Diseases Collaborators, 2024; Salvador-Erro et al., 2024). The acquisition of virulence factors (VFs) enables *E. coli* to cause diarrheal diseases and, in severe cases, extra-intestinal infections that may lead to life-threatening outcomes (Nhambirre et al., 2025; Akhtar et al., 2024; Jones et al., 2024).

The remarkable diversity of pathogenic *E. coli* has led to its classification into two primary categories: extra-intestinal pathogenic *E. coli* (ExPEC) and diarrheagenic *E. coli* (DEC). Infections caused by DEC are frequently characterized by watery, bloody, or mucous diarrhea, often accompanied by abdominal cramps, and may present with symptoms such as fever, nausea, or, in severe cases, more critical clinical manifestations (Black et al., 2024). Based on distinct VF profiles and host pathological features, DEC is further divided into six pathogenic types: enteropathogenic *E. coli* (EPEC), enterotoxigenic *E. coli* (ETEC), enteroinvasive *E. coli* (EIEC), enterohemorrhagic *E. coli* (EHEC), enteroaggregative *E. coli* (EAEC), and diffusely adherent *E. coli* (DAEC) (Kjellin et al., 2024).

Among these, EAEC has emerged as a particularly complex and concerning diarrheagenic pathogen, responsible for acute and persistent diarrhea across diverse populations, including children, adults, and travelers. It plays a significant role in contributing to growth retardation in children, particularly in low-income or developing countries (Dereje et al., 2024; Jones et al., 2024; Black et al., 2024; Kim et al., 2022). A defining characteristic of EAEC is its unique ability to adhere to HEp-2 cells in a “stacked bricks” pattern, a feature that distinguishes it from other pathogenic types (Sheikh et al., 2022). However, due to the labor-intensive nature of adhesion assays, molecular PCR-based techniques are widely used to detect specific virulence genes, facilitating the identification of EAEC strains (Jones et al., 2024; Black et al., 2024; Kjellin et al., 2024; Kim et al., 2022; Sheikh et al., 2022; Wang et al., 2021).

Globally, EAEC holds substantial epidemiological importance, imposing a considerable disease burden, particularly in developing regions (Rousham et al., 2021). Despite this, the intricate epidemiology of EAEC has rendered its outbreaks and pathogen characteristics poorly documented on a global scale (Wang et al., 2021; Lee et al., 2024). Furthermore, outbreak management literature often lacks adequate evidence to trace infection sources, and there remains an absence of comprehensive guidelines for conducting tracing investigations and molecular source tracking (Rousham et al., 2021; Lee et al., 2024; Akhtar et al., 2023).

In certain regions of China, disparities in economic development and inadequacies in basic healthcare infrastructure have exacerbated the risk of childhood diarrhea, particularly in vulnerable populations (Li et al., 2024). Longitudinal surveillance data collected from acute diarrhea patients in China between 2009 and 2018 have revealed that EAEC exhibits the highest detection rate among all DEC types (Yuan et al., 2024). This trend is most pronounced in children residing in rural areas and adults living in urban centers (Liu et al., 2024). Despite its prevalence, EAEC remains underrepresented in the literature and often overlooked by researchers, highlighting a significant gap in understanding its public health impact (Jiang et al., 2023).

This study presented a retrospective analysis of a foodborne diarrheal outbreak caused by EAEC in a county school in Shandong Province, China, in 2023. Advanced molecular techniques, including pulsed-field gel electrophoresis (PFGE) and whole-genome sequencing (WGS), were employed to trace the isolated strains. Phylogenetic analyses and comparative genomic assessments uncovered potential pathogenic genetic factors and shed light on the food safety risks posed by EAEC. Furthermore, the study delved into antimicrobial resistance profiles and elucidated transmission pathways of the isolated strains, offering critical insights to inform early warning systems and enhance antibiotic stewardship efforts.

## Materials and methods

### Description of the outbreak and epidemiological investigation

On June 15, 2023, a clustered outbreak of acute diarrhea was reported in schools located in a county of Shandong Province, China. In response, the Shandong Provincial Center for Disease Control and Prevention (Shandong CDC) swiftly initiated an on-site epidemiological investigation. Suspected cases were defined as individuals exhibiting at least one of the following symptoms within a short timeframe: nausea, vomiting, or diarrhea, accompanied by abdominal pain, fever, bloating, or headache. All individuals meeting the suspected case definition were included in the investigation. Confirmed cases were those whose stool specimens tested positive for EAEC during laboratory analysis.

To identify the origin of the outbreak and assess the scope of infection, face-to-face and telephone epidemiological interviews were conducted with all 1,215 individuals present at the school. These interviews covered the school’s sanitation and environmental conditions, the students’ symptoms and medical treatments, and their food and water consumption patterns at school. Samples (*n* = 91) from symptomatic patients, asymptomatic carriers, food items, and environmental water sources were collected for laboratory testing. A case–control study was conducted to evaluate potential risk factors. Additionally, a questionnaire survey was distributed to both suspected cases and individuals who did not exhibit symptoms during the investigation period. Ethical approval for this study was obtained from the Ethics Review Committee of the Shandong Provincial CDC before the study commenced.

### Sample collection and bacterial identification

To rapidly identify the source of the outbreak, we implemented a multi-dimensional sampling strategy and comprehensively collected four types of samples: (1) Fresh fecal samples collected using ESwabs (Copan) from 20 canteen employees involved in cooking and dining services, as well as from 16 newly affected students with diarrhea who had either received no antibiotic treatment or treatment lasting ≤24 h. (2) Water samples from 18 drinking water collection points (including 14 direct drinking water terminals and 4 water nodes in canteen kitchens) covering key areas such as teaching zones, dormitories, and canteens. Each sample (500 mL) was aseptically collected into dedicated sampling bags following sterile procedures. (3) Food samples, including refrigerated livestock and poultry meat products (*n* = 11), aquatic products (*n* = 5), raw fruits and vegetables (*n* = 10), cured meats and vegetables (*n* = 7), and condiments exposed to the external environment (*n* = 3). (4) Vector samples consisting of 20 adult flies collected from the canteen’s external environment. All food and vector samples were sealed in sterile sampling bags for preservation. All samples were kept in cool boxes with ice packs (4–8°C) upon collection and were transported to the laboratory at the end of the sampling day.

Furthermore, in this epidemic traceability investigation, we first implemented a standardized pathogen screening program for the 36 fecal samples collected: using a multiplex nucleic acid detection kit for diarrheal syndrome, we simultaneously tested for 12 common diarrheal pathogens, specifically including *Vibrio cholerae*, *Vibrio parahaemolyticus*, *Salmonella*, *Shigella*, DEC (EPEC, EIEC, ETEC, EAEC, EHEC), *Campylobacter* spp., *Yersinia enterocolitica*, *Clostridioides difficile*, *Cronobacter sakazakii*, *Aeromonas hydrophila*, *Plesiomonas shigelloides*, and *Vibrio fluvialis.*

After initial screening positive for EAEC. Stool samples were inoculated onto MacConkey Agar (MAC) medium, while drinking water samples were filtered using the membrane filtration technique, with the membranes subsequently placed on MAC medium. Food and fly samples (aseptic grinding) underwent enrichment in EC broth before inoculation onto MAC medium. After all media were incubated at 36°C for 24 h, 5–10 typical suspected colonies were picked and screened for EAEC colonies using five multiplex nucleic acid detection kits for diarrheagenic *Escherichia coli*. Species identification of EAEC positive colonies was performed using a matrix-assisted laser desorption ionization-time of flight mass spectrometer (MALDI-TOF MS). Finally, samples were preserved in 30% glycerol broth and stored at −80°C for future analyses.

### Antimicrobial susceptibility testing

Antimicrobial susceptibility testing was performed for 15 antibiotics, including imipenem, cefotaxime, ceftazidime, cefoperazone, amoxicillin/clavulanic acid, piperacillin/tazobactam, gentamicin, amikacin, ciprofloxacin, trimethoprim/sulfamethoxazole, tetracycline, florfenicol, polymyxin B, nitrofurantoin, and tigecycline. The broth microdilution method was employed in strict accordance with the guidelines outlined by the Clinical and Laboratory Standards Institute (CLSI). *E. coli* ATCC 25922 served as the quality control strain to ensure the reliability and accuracy of the susceptibility testing.

### PFGE analysis

PFGE was utilized as a rapid and effective method to assess the genetic relatedness among strains, making it an indispensable tool in molecular epidemiological investigations of foodborne outbreaks. PFGE fingerprinting of 15 isolated strains was conducted following the standardized protocols recommended by PulseNet USA (CDC). The restriction enzyme *XbaI* was used to digest DNA from the isolated strains and the reference strain H9812. The resulting DNA fragments were separated and analyzed using a PFGE system. BioNumerics 8.0 software was employed for fingerprint processing and cluster analysis to establish genetic relationships between the strains.

### WGS

Genomic DNA from the outbreak isolates was extracted using the Centra Puregene Yeast/Bact. Kit (QIAGEN, Hilden, Germany). All isolated strains were subjected to second-generation sequencing using the Illumina HiSeq platform, with an average coverage of 100x. The outbreak cluster representative isolated strain BZ21 and the pan-drug-resistant isolated strain BZ01 were selected and subjected to third-generation sequencing through the PacBio platform to confirm and supplement the results of second-generation sequencing. The PacBio reads achieved N50 of 17–18 kb. The quality of raw sequencing reads was evaluated using FastQC 0.12.1. Raw reads were quality-filtered and trimmed using Trimmomatic 0.39 before assembly, and *de novo* assembly into contigs was performed using SPAdes 3.15.2. The assembled genomes were further assessed with Quast 5.2.0 to evaluate key assembly parameters. Genome annotation was conducted with Prokka 1.14.6. For specific parameters, please refer to the supplementary document (Supplementary Table S1).

Obtaining multilocus sequence typing (MLST) based on PubMLST database.^1^ VFs were identified using the Virulence Factor Database (VFDB). Serotypes and antimicrobial resistance genes were analyzed with SerotypeFinder 2.0.2 and ResFinder 4.6.0, respectively. The presence of plasmids was predicted using PlasmidFinder 2.1.6, while insertion sequences (ISs) were identified through the ISfinder database.

Comparative genomic analyses were conducted using the BLAST Ring Image Generator (BRIG) to generate genomic ring plots of outbreak-associated isolates, facilitating the comparison of multiple whole-genome sequences and the localization of annotated genes. Genetic background maps surrounding antibiotic resistance genes were created with Easyfig. All genome assemblies generated during this study have been deposited in GenBank for public access (SAMN46142811–SAMN46142825).

### Phylogenetic analysis

To expedite the identification of the infection source and enhance laboratory evidence, multiple analytical methods were employed to investigate the genetic characteristics of the outbreak isolates. This study focuses on the evolutionary tracing of EAEC outbreak strains, utilizing the internationally recognized EAEC reference strain 042 (GCA_000027125.1) as the reference genome to minimize homologous alignment biases caused by using distantly related reference genomes. Furthermore, the *astA*, *aggR*, and *pic* genes serves as the characteristic gene of EAEC, comparing them with the whole-genome sequences of *E. coli* strains available in the NCBI database. A total of 165 whole-genome sequences carrying EAEC-specific virulence genes were identified. As highlighted by Erick Denamur (Al-Mir et al., 2021), *E. coli* exhibits a robust phylogenetic structure comprising nine distinct phylogroups: A, B1, B2, C, D, E, F, G, and H.

To elucidate the phylogenetic relationships, a maximum-likelihood phylogenetic tree was constructed using 34 whole-genome sequences from the nine phylogroups, 165 global *E. coli* strains harboring EAEC-specific virulence genes, and 15 EAEC isolates from the current outbreak. Single nucleotide polymorphism (SNP) calling was conducted using Snippy 4.6.0, and recombinant variants were excluded using Gubbins 3.4 to ensure the analysis relied solely on recombination-free SNPs. The phylogenetic tree was generated with IQ-TREE. The GTR + G + I model and 1,000 bootstrap repetitions were adopted to improve statistical robustness, and visualized using iToL to facilitate interpretation (Supplementary Table S3).

### Adhesion assay

Adhesion assays were performed to evaluate the bacterial adhesion patterns of the 15 isolated strains. Monoclonal bacterial colonies were initially inoculated into liquid LB medium and incubated overnight at 37°C with shaking. The following day, the cultures were subcultured into fresh LB medium and grown with shaking until an OD_600_ of 0.6 was achieved. Bacterial cells were then resuspended in serum-free DMEM (Dulbecco’s Modified Eagle Medium containing 1% D-mannose) for subsequent experiments.

HEp-2 cells, seeded at a density of 1 × 10^5^ cells per well into 24-well plates pre-coated with coverslips, were incubated for 24 h at 37°C in a 5% CO₂ atmosphere. After reaching approximately 80% confluency, the cell plates were washed three times with DMEM, and a mixture of 900 μL of DMEM containing 1% D-mannose and 100 μL of bacterial suspension (6 × 10^8^ CFU/mL) was added to each well. The cultures were incubated at 37°C for 3 h. Following incubation, the wells were washed three times with phosphate-buffered saline (PBS) to remove non-adherent bacteria.

To fix the adherent cells, 1 mL of pre-chilled anhydrous ethanol was added to each well, and the plates were incubated at −20°C for 10 min. After three additional washes with PBS, the cells were stained with Giemsa stain for 30 min, washed with sterile water, and fixed with methanol before mounting. The adhesion patterns of the bacteria were subsequently observed and categorized under a microscope (Olympus, CKX53, Japan).

## Results

### Epidemiological investigation of the diarrheal outbreak

A case search was conducted among students, faculty, and canteen staff based on the established case definition, identifying 44 suspected cases: 42 students and two teachers. No related diarrhea symptoms were found among the canteen staff. Cases were distributed across all grade levels, with a demographic composition of 24 males and 20 females, resulting in a male-to-female ratio of 1.2:1. The predominant clinical manifestations included abdominal pain (43 cases, 97.73%), diarrhea (42 cases, 95.45%), and watery stools (26 cases, 59.09%), often accompanied by nausea (15 cases, 34.09%), vomiting (five cases, 11.36%), and fever (four cases, 9.09%).

**Figure 1:**
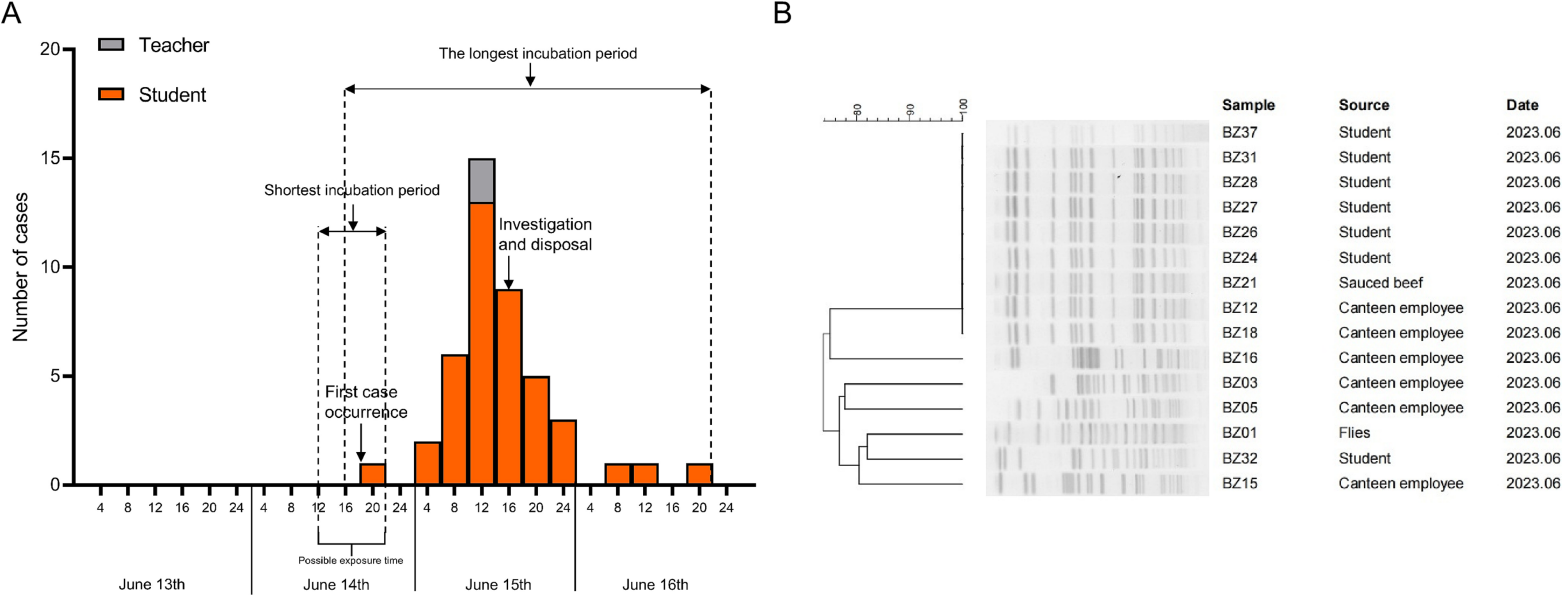
Investigation of outbreak epidemics. **(A)** Epidemic curve for outbreak cases and timeline of key events in the investigation. **(B)** PFGE patterns of the EAEC strains isolated from individuals involved in the outbreak.

The outbreak’s onset was marked by the first case at 8:00 PM on June 14, followed by a rapid surge in similar cases. Within 24 h, 38 individuals developed symptoms, accounting for 86.36% of the total cases. By 8:00 PM on June 15, the number of new cases had significantly declined. The average incubation period was calculated to be 26 h, ranging from 8 to 52 h, consistent with the epidemiological profile of a point-source outbreak (Figure 1A).

The investigation revealed that the school employed a direct drinking water purification system, maintained regularly, to supply hot and warm water. The hot water terminals were equipped with an automatic shutoff mechanism to prevent dispensing when temperatures fell below 100°C. The school operated under a closed management system, and its cafeteria, contracted to an external food company, provided meals for all students and staff. On-site inspections uncovered several hygiene deficiencies in the cafeteria. While sanitation was deemed average, the floors were damp, and mosquitoes and flies were observed. Food preparation practices raised concerns, with food processing tools lacking clear labeling to distinguish between raw and cooked items. Additionally, improper storage practices were noted, including instances of raw and cooked foods being stored together, further highlighting potential sources of contamination.

### Epidemiological investigation and analysis

The findings from the epidemiological survey indicated that the likelihood of transmission via respiratory routes or drinking water was minimal. However, a potential risk for exposure was identified due to sanitation issues within the cafeteria. Based on these observations, it was hypothesized that the consumption of contaminated food could be the primary exposure factor. To test this hypothesis, a case–control study was conducted, involving 44 suspected cases as the case group and 89 asymptomatic individuals randomly selected as the control group. The investigation focused on food consumption patterns in the cafeteria between June 13 and June 15. The results revealed that the cold beef with sauce served for lunch on June 14 was a significant risk factor for illness, with an odds ratio (OR) of 6.943 (95% CI: 2.891–16.676), and this difference was found to be statistically significant (Table 1).

**Table 1:** Association of illness with food items.

Date	Food	Exposed population/[*n*(%)]		Chi-squared value	*p*-value	Odds ratio (95%CI)
		Case group (*n* = 44)	Control group (*n* = 89)			
Breakfast on June 13th	Tea egg	18 (40.91)	39 (43.82)	0.102	0.75	0.888 (0.427–1.846)
	Rougamo	37 (84.09)	62 (69.66)	3.221	0.073	2.302 (0.912–5.809)
	Fried meat with cauliflower	18 (40.91)	36 (40.45)	0.003	0.959	1.019 (0.489–2.126)
Lunch on June 13th	Marinated chicken leg	22 (50.00)	39 (43.82)	0.453	0.501	1.282 (0.621–2.645)
	Fried shrimps	21 (47.73)	39 (43.82)	0.182	0.67	1.171 (0.567–2.416)
	Braised Spanish mackerel	24 (54.55)	61 (68.54)	2.5	0.114	0.551 (0.262–1.158)
	Chili and sour potato	31 (70.45)	73 (82.02)	2.311	0.128	0.523 (0.225–1.215)
	Rice	18 (40.91)	46 (51.69)	1.37	0.242	0.647 (0.312–1.344)
	Steamed bun	5 (11.36)	18 (20.22)	1.616	0.204	0.506 (0.174–1.467)
Dinner on June 13th	Braised eggplant with minced meat	9 (20.45)	28 (31.46)	1.776	0.183	0.560 (0.237–1.322)
	Scrambled eggs with green pepper	4 (9.09)	18 (20.22)	2.644	0.104	0.394 (0.125–1.247)
	Stewed pork slices	33 (75)	77 (86.52)	2.73	0.098	0.468 (0.187–1.166)
	Fried bean curd bamboo with chili	15 (34.09)	29 (32.58)	0.03	0.862	1.070 (0.498–2.299)
	steamed roll	20 (45.45)	28 (31.46)	2.5	0.114	1.815 (0.863–3.817)
	Mixed fried rice	13 (29.55)	29 (32.58)	0.126	0.723	0.868 (0.396–1.902)
Breakfast on June 14th	Steamed pumpkin	4 (9.09)	7 (7.87)	0.058	0.809	1.171 (0.324–4.236)
	Scallion pancake	30 (68.18)	57 (64.04)	0.223	0.637	1.203 (0.558–2.593)
	Fish-flavored pork	28 (63.64)	66 (74.16)	1.573	0.21	0.610 (0.281–1.325)
Lunch on June 14th	Garlic pork	8 (18.18)	28 (31.46)	2.63	0.105	0.484 (0.199–1.176)
	Sauced beef	36 (81.82)	35 (39.33)	21.363	<0.001	6.943 (2.891–16.676)
	Bone and Meat Harmony	32 (72.73)	73 (82.02)	1.531	0.216	0.584 (0.248–1.376)
	Stir-fried tofu skin with chives	14 (31.82)	34 (38.20)	0.52	0.471	0.755 (0.351–1.622)
	Vegetable Steamed flower roll	5 (11.36)	22 (24.72)	3.246	0.072	0.390 (0.137–1.114)
	Hand-pulled flatbread	29 (65.91)	68 (76.40)	1.643	0.2	0.597 (0.270–1.319)
Dinner on June 14th	Braised pork belly with diced potatoes	30 (68.18)	47 (52.81)	2.854	0.091	1.915 (0.897–4.089)
	Moo shu pork with cucumber	15 (34.09)	22 (24.72)	1.288	0.256	1.575 (0.717–3.463)
	Stir-fried Spinach	2 (4.55)	7 (7.87)	0.514	0.473	0.558 (0.111–2.804)
	Stir-fried pork with winter melon	15 (34.09)	30 (33.71)	0.002	0.965	1.017 (0.474–2.181)
	Plain steamed flower bun	9 (20.45)	9 (10.11)	2.691	0.101	2.286 (0.836–6.249)
	Two-tone roll	8 (18.18)	14 (15.73)	0.128	0.72	1.190 (0.458–3.094)
Breakfast on June 15th	Carotene-filled bun	11 (25)	14 (15.73)	1.658	0.198	1.786 (0.734–4.346)
	Cabbage and Pork Bun	38 (86.36)	78 (87.64)	0.043	0.836	0.893 (0.307–2.598)
	Garlic stir-fried romaine lettuce	31 (70.45)	59 (66.29)	0.233	0.629	1.213 (0.554–2.652)

Odds ratio (OR) and 95% CI, and *p* values are shown.

### Laboratory investigation

In an effort to identify the pathogen responsible for the outbreak, strain isolation and biochemical identification were performed on biological samples (*n* = 91) collected from fecal matter (36 copies), food (36 copies), water (18 copies), and environmental sources (1 copies). A total of 15 isolates were identified as EAEC, all of which tested positive for the *astA* characteristic gene. Of these, seven strains were isolated from the feces of diarrheal students, six from the feces of cafeteria staff, one from cold-stored braised beef with sauce, and one from flies in the canteen’s external environment.

### PFGE molecular traceability

PFGE is a widely used genetic typing technique for pathogen outbreak investigations, providing valuable insights into the origin of outbreak strains. In this study, after restriction enzyme digestion with *XbaI*, the 15 isolated EAEC strains displayed seven distinct PFGE patterns, with similarities ranging from 78.22 to 100%. Notably, nine of the isolates (six from diarrheal students, two from cafeteria staff, and one from the cold-stored braised beef with sauce) shared an identical PFGE pattern with 100% similarity (Figure 1B). This suggested that these isolates were clonal variants, exhibiting a high degree of genetic similarity, and it further confirmed that the outbreak of EAEC infection was caused by the consumption of cold sauced beef contaminated by the Canteen employee.

### Genome analysis and phylogenetic comparison of EAEC group isolates

To gain a precise understanding of the population structure and geographic origins of the isolates associated with this diarrheal outbreak, our study integrated the genomes of the outbreak isolates with 34 whole-genome sequences from the nine phylogenetic groups of *E. coli* and 165 whole-genome sequences of EAEC strains from global datasets. A phylogenetic tree was constructed, revealing that the genome sizes of the outbreak isolates ranged from 4,719,483 bp to 5,155,634 bp, distributed across three phylogenetic groups: A, B2, and D. SNP analysis of the 214 strains identified 3,826 SNPs in total. Notably, the nine ST394 isolates from this outbreak formed a tightly clustered group, with their core genomes differing by only 0 to 5 SNPs. This finding strongly indicated that these nine EAEC isolates originated from the same clone and exhibited a high degree of genetic homology, classifying them within the D phylogenetic group. Importantly, the results of both PFGE and WGS corroborated these findings, confirming the remarkably close genetic relationship among the nine outbreak-related isolates. Combined with epidemiological evidence, it was determined that this outbreak was triggered by students consuming cold sauced beef contaminated by Canteen employee, which led to the EAEC diarrhea outbreak.

**Figure 2:**
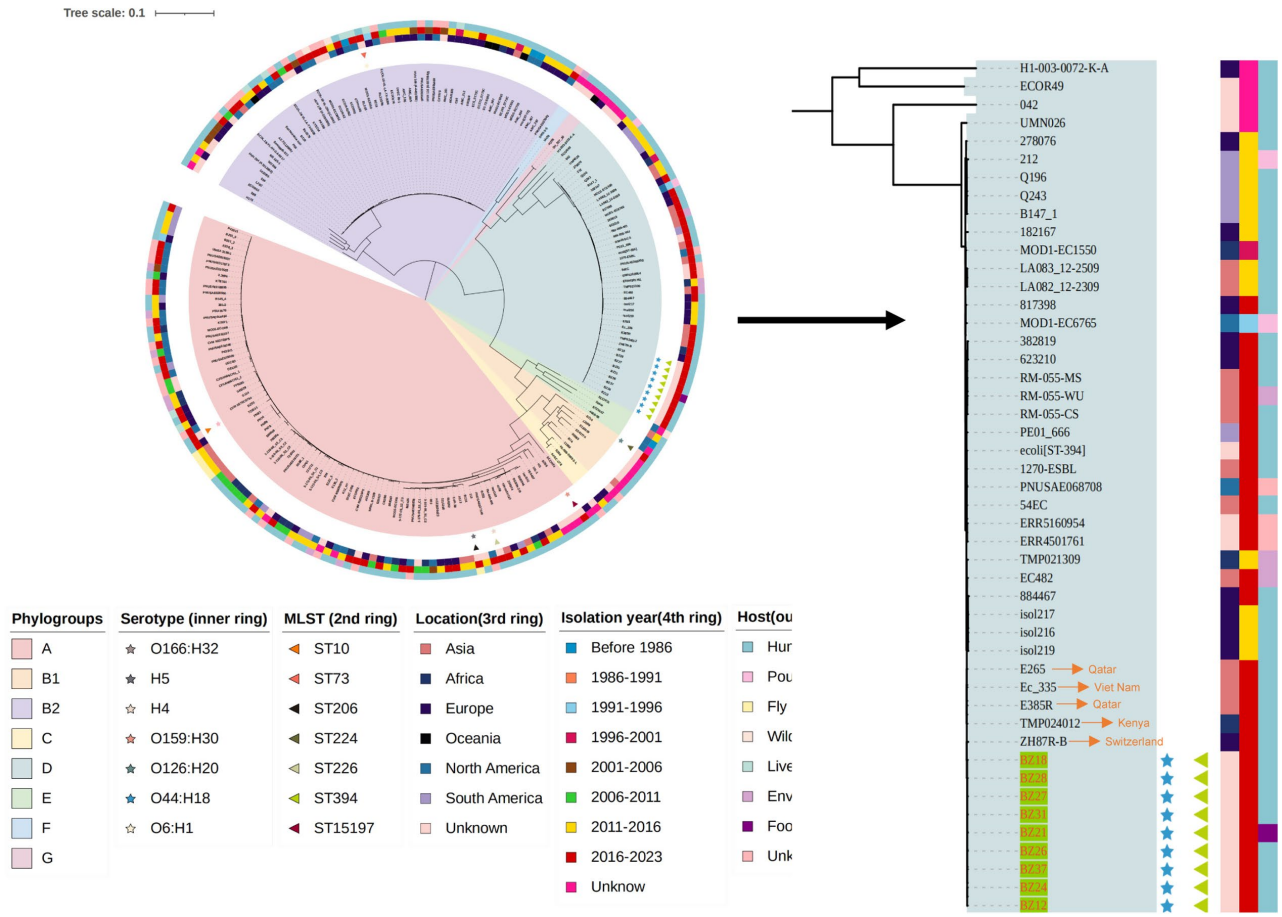
The phylogenetic tree of the 15 EAEC group isolates this study and another 199 EAEC group isolates in the NCBI database.

Additionally, phylogenetic analysis revealed that the outbreak cluster isolates belonged to the same sub-branch as 34 EAEC isolates from Asia, Africa, Europe, Oceania, North America, and South America. Within this evolutionary branch, five isolates were found to be closely related to the outbreak cluster strains. These included the Swiss strain ZH87R-B (2019, biological sample: SAMN13944199), the Kenyan strain TMP024012 (2019, biological sample: SAMEA5611987), the Vietnamese strain Ec_335 (2018, biological sample: SAMEA6813705), and two Qatari strains (2018, biological samples: SAMN13829841 and SAMN13829791), all of which were isolated from rectal swabs (Figure 2).

### Virulence gene patterns and HEp-2 cell assay

To investigate the virulence gene profiles of the 15 isolated strains, we utilized the whole-genome sequence of strain BZ21, obtained through third-generation sequencing, as a reference to construct circular genomic maps using the BRIG. The analysis revealed that the genomic sequences of most isolates closely aligned with the reference strain, particularly strains BZ12, BZ18, BZ21, BZ24, BZ26, BZ27, BZ28, BZ31, and BZ37. These isolates exhibited strong phylogenetic relationships and consistent virulence gene patterns.

**Figure 3:**
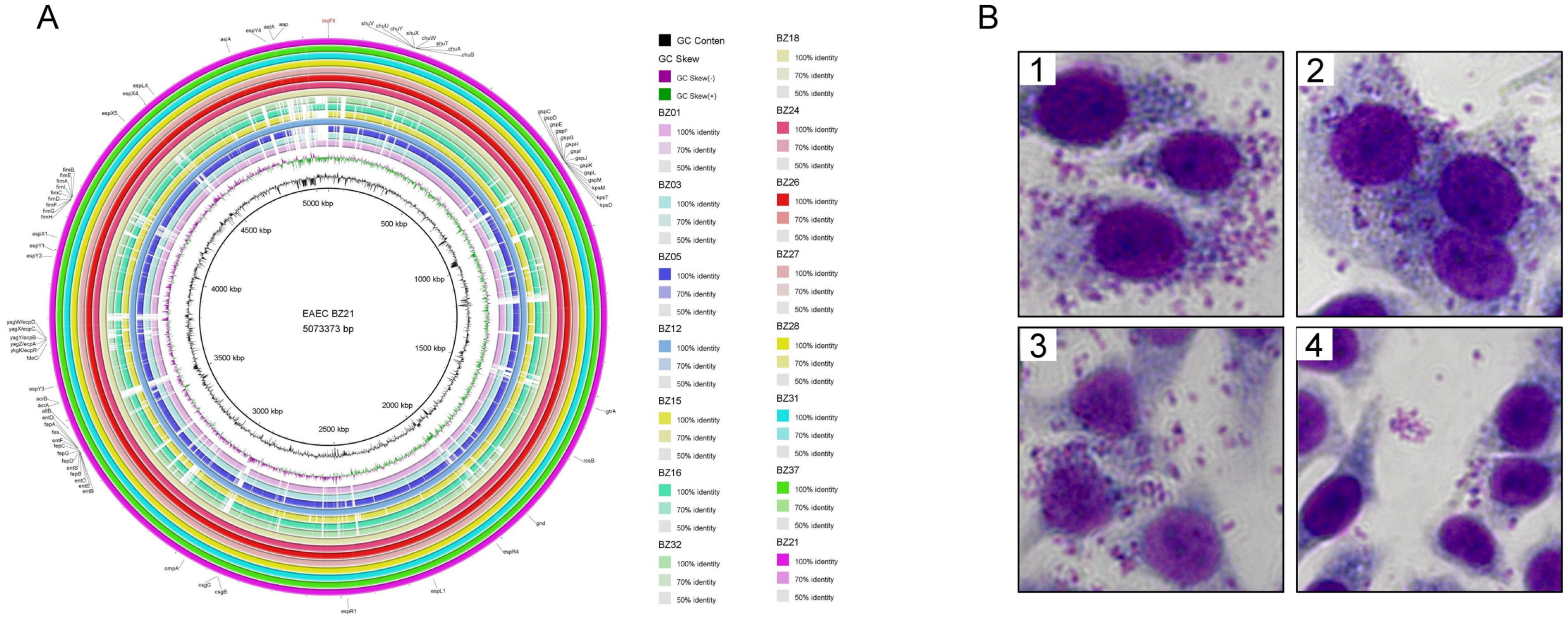
Pathogenicity of outbreak isolates. **(A)** BLAST atlas produced using BLAST ring image generator. Comparative BLASTn analysis with 70, 90, and 100% identities are displayed, and gaps in circles represent regions with no identity of genes. The innermost ring represents the EAEC BZ21 used as the reference and its coordinates. The second ring (in black) plots the GC content of the reference, followed by its GC skew (in purple/green). The following rings represent isolated EAEC (in order from inside out: BZ01, BZ03, BZ05, BZ12, BZ15, BZ16, BZ32, BZ18, BZ24, BZ26, BZ27, BZ28, BZ31, BZ37, and BZ21). Outer ring annotation: black, virulence genes; red, plasmid replicons. **(B)** Adherence phenotype to Hep-2 cells of different EAEC strains. B1 BZ21 (AA), B2 BZ12 (AA), B3 BZ24 (AA), B4 BZ15 (LA).

WGS identified a total of 72 virulence genes in the nine outbreak-associated isolates, with 70 located on the chromosome and two on plasmids. These genes were primarily associated with key VFs, including adhesins (*fimA-I* and *fdeC*), iron acquisition systems (*kpsD*, *kpsM*, and *kpsT*), type III secretion system effectors (*espR*, *espL*, *espY*, and *espX*), heat-stable enterotoxin 1 (*astA*), and the dispersin gene (*aap*). Additionally, the circular genomic map revealed partial deletions and sequence variations relative to the reference genome, highlighting genomic diversity within the isolates (Figure 3A; Supplementary Figure S1).

HEp-2 cell adhesion assays were conducted using strains BZ21, BZ12, and BZ24, which were sourced from different outbreak-related origins (sauced beef, canteen employee, and student, respectively), as well as strain BZ15, which was phylogenetically distant from the outbreak cluster. The results demonstrated that strains BZ21, BZ12, and BZ24 exhibited aggregative adherence (AA), whereas strain BZ15 displayed localized adherence (LA) (Figure 3B). These findings suggested that strains carrying the *astA* virulence gene might exhibit variable adhesion patterns, with AA being the predominant type. This observation aligned with previous studies (Helalat et al., 2020), further supporting the association between *astA* and specific adherence phenotypes.

### Phenotypic and genotypic antibiotic resistance

**Figure 4:**
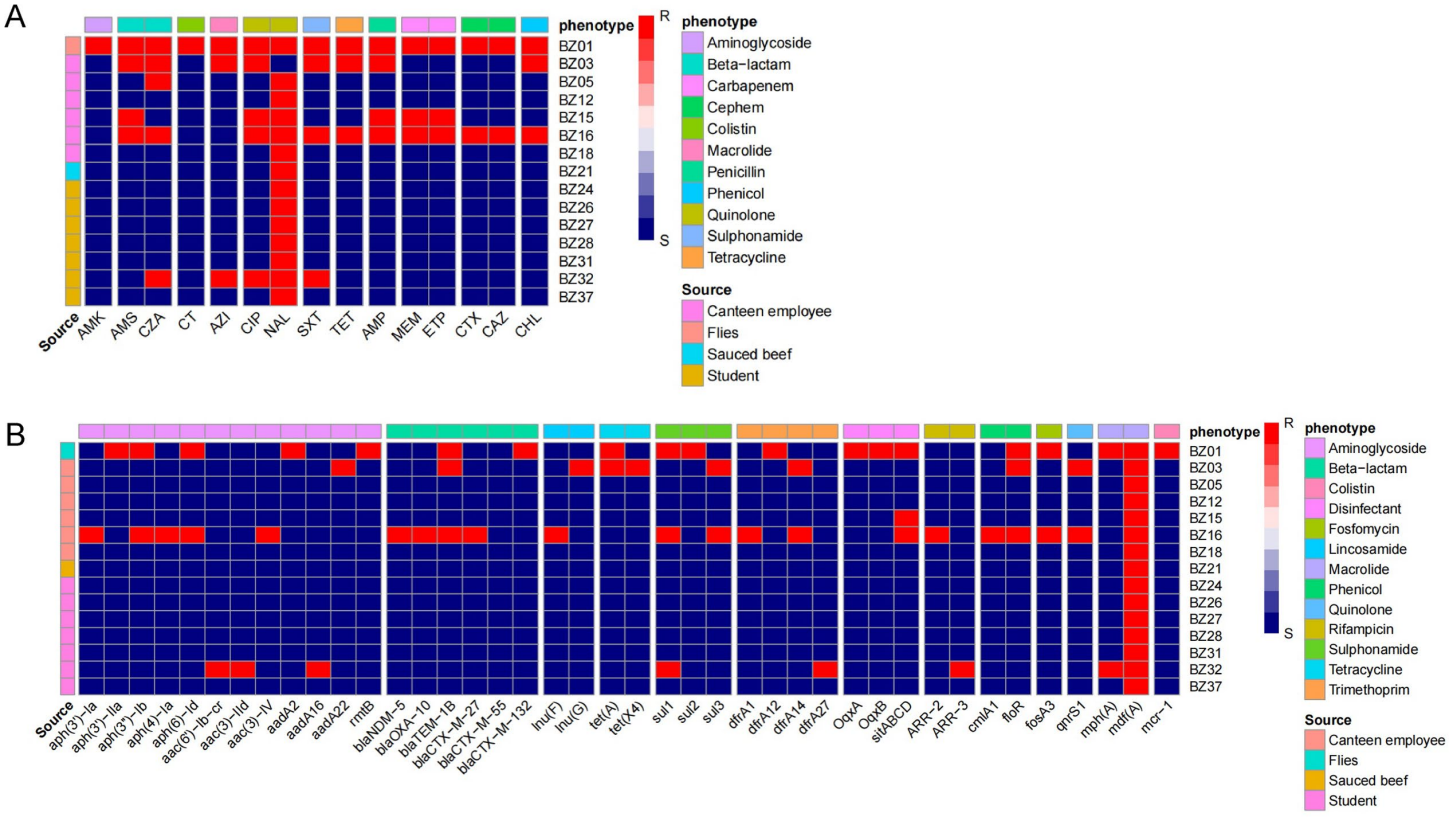
Drug resistance in outbreak isolates. **(A)** Phenotype of drug resistance. MICs of 15 antimicrobials belonging to 11 antimicrobial classes were tested. **(B)** Research on drug resistance genes. The resistance (red block) and susceptibility (blue block) of isolates to the antibiotics were indicated using different colors.

To elucidate the antimicrobial resistance profiles of the isolates implicated in this outbreak and provide accurate clinical guidance, we investigated both the phenotypic resistance and the genotypic determinants of antimicrobial resistance. The analysis revealed that nalidixic acid exhibited the highest resistance rate, with 93.33% (14/15) of the isolates demonstrating resistance. Notably, while all outbreak cluster isolates exhibited the nalidixic acid resistance phenotype, they remained susceptible to all other tested antibiotics. However, isolates BZ16, BZ03, BZ15, and BZ32 were identified as multidrug-resistant (MDR) strains, showing high resistance rates to ciprofloxacin (100%, 4/4), ampicillin/sulbactam (75%, 3/4), ceftazidime/avibactam (75%, 3/4), and co-trimoxazole (75%, 3/4) (Figure 4A). Remarkably, an extensively drug-resistant (XDR) strain, BZ01, was isolated from flies. This strain exhibited resistance to all 15 antibiotics tested, highlighting its potential threat to public health.

WGS further identified 41 resistance-associated genes spanning 13 classes of antimicrobials. All outbreak-related strains carried the *mdf(A)* gene, conferring resistance to macrolides. Importantly, the non-outbreak-related strain BZ01 harbored the *mcr-1* gene, associated with polymyxin resistance. Additionally, BZ16, BZ01, and BZ03 were found to carry extended-spectrum *β*-lactamase (ESBL) genes, including *bla*_NDM-5_, *bla*_TEM-1B_, *bla*_CTX-M-27_, *bla*_CTX-M-55_, *bla*_CTX-M-132_, and *bla*_OXA-10_ (Figure 4B).

This study analyzed the association between antibiotic resistance phenotypes and genotypes in EAEC isolates using a correlation matrix. The results revealed significant correlations between multiple resistance genes and specific antibiotics: The *rmtB*, *aadA2*, and *aph (3′)-IIa* genes all directly mediate resistance to amikacin (AMK) aminoglycoside antibiotics. The *floR* gene showed a perfect correlation (coefficient = 1) with tetracycline (TET), and the *sitABCD* gene exhibited a perfect correlation (coefficient = 1) with meropenem (MEM). Additionally, we observed a correlation coefficient of −1 between *aac(3)-IId* and nalidixic acid (NAL), suggesting that the presence of this gene may be associated with increased sensitivity of the strains to NAL. However, the underlying biological mechanisms require further validation (Supplementary Figure S2).

### Genetic structure analysis of plasmids carrying *bla*_CTX-M-132_ and *mcr-1*

The XDR isolate BZ01 was forecast to carry three plasmids (PLBZ01-1, PLBZ01-2, and PLBZ01-3) and three plasmid replicons (IncFIB, IncI2, and IncN). Detailed genetic analysis of the PLBZ01-2 genomic sequence, which harbored both *bla*_CTX-M-132_ and *mcr-1* resistance genes, belonged to the IncI2 incompatibility group. This plasmid possessed a characteristic backbone responsible for replication, maintenance, and horizontal transfer. Comparative analysis showed that the PLBZ01-2 backbone shared 98% BLAST query coverage and 99.64% nucleotide identity with the previously reported plasmid pM-132-4972 (GenBank accession no. MT773671.1), which was isolated in Zhejiang, China, in 2019.

**Figure 5:**
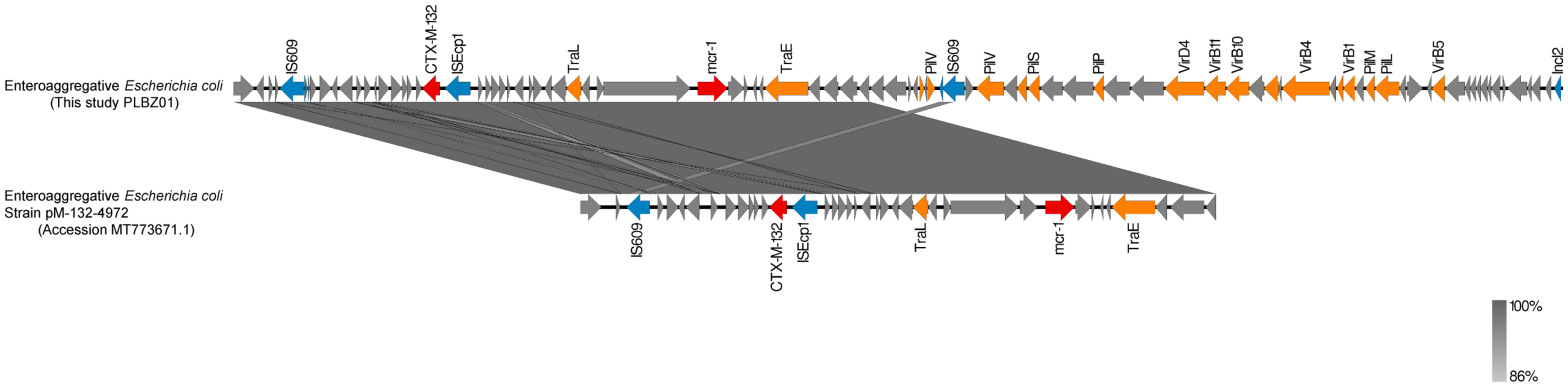
Genetic analysis of plasmid sequences of PLBZ01-2 (this study, BZ01) and pM-132-4972 (MT773671.1). 99% homology are marked by gray shading. Arrows represent direction of transcription. Red open reading frames (ORFs) indicate mcr-1/CTX-M-132, blue ORFs indicate insertion elements, orange ORFs indicate mobile elements, and gray ORFs indicate other proteins or proteins of unknown function.

Both plasmids were found to encode *bla*_CTX-M-132_ and *mcr-1*, along with ISs, transposons, and conjugation-related *Tra* gene families, indicating a high level of genetic similarity. Furthermore, the PLBZ01-2 plasmid contained genes encoding two types of pili (*Vir* and *pil* operons), which might facilitate horizontal gene transfer via plasmid interaction, homologous recombination, and transposon-mediated mechanisms. Of particular interest, an *ISEcp1* gene (1,262 bp, IS1380 family) was identified 253 bp upstream of *bla*_CTX-M-132_. This finding aligned with previous studies suggesting that *ISEcp1* plays a significant role in the mobilization and expression of *bla*_CTX-M-132_, underscoring its relevance in plasmid-mediated antibiotic resistance (Figure 5).

## Discussion

This study provided a comprehensive investigation into an outbreak of acute gastroenteritis in a school setting, ultimately identifying the etiology as a foodborne illness caused by EAEC infection. The clinical profile of affected individuals was dominated by abdominal pain and diarrhea, often accompanied by nausea, vomiting, and fever. The temporal distribution of cases, with a peak incidence between 8:00 AM and 12:00 PM on June 15, displayed a classic single-peak epidemic curve. This pattern, characterized by a concentrated onset time, strongly suggested a point-source exposure.

Epidemiological evidence from the case–control study pinpointed the implicated meal as lunch served on June 14, with cold beef with sauce identified as the likely vehicle of transmission. This conclusion was further supported by the temporal alignment between the suspected food consumption and the exposure period indicated by the epidemic curve. The rapid containment of the outbreak following the closure of the school cafeteria, isolation of cafeteria staff, and comprehensive disinfection efforts provided additional confirmation of the foodborne origin. The association between the contaminated food item and the outbreak highlighted the critical importance of stringent food safety practices, particularly in institutional settings.

To rapidly and accurately trace the source of infection and analyze the genetic evolutionary relationships and population structure of the causative pathogens, this study employed PFGE to confirm the outbreak. The PFGE results were further complemented with high-resolution WGS, providing a deeper and more reproducible insight into the outbreak’s genetic context. Genomic circular plots and phylogenetic analyses revealed a striking genetic similarity among EAEC strains isolated from the contaminated beef with sauce, infected individuals, and asymptomatic canteen employees. The genetic variation within the outbreak cluster was minimal, ranging from 0 to 5 SNPs, providing robust molecular evidence that corroborated the epidemiological findings. These results decisively linked the outbreak to the contamination of beef with sauce by asymptomatic canteen employees during food preparation, either through direct handling or indirect contact. A critical finding of this study was the role of asymptomatic carriers in the outbreak, demonstrating that asymptomatic individuals infected with EAEC could serve as reservoirs of infection and pose significant risks to the broader population. This observation aligned with the conclusions of Smith et al., reinforcing the public health implications of asymptomatic carriers in foodborne outbreaks (Smith et al., 2007).

Notably, the 15 EAEC strains identified in this study were classified into six sequence types (STs), with one strain remaining untyped. Nine of the outbreak-related strains were assigned to ST394, a lineage that demonstrated substantial environmental adaptability. The coexistence of multiple STs within the outbreak highlighted the genetic diversity of these strains, which likely contributed to their survival and resilience under external environmental pressures. This genetic heterogeneity underscored the necessity of stringent food hygiene practices and regular surveillance for pathogenic bacteria to mitigate the risk of foodborne outbreaks, particularly in institutional settings such as schools.

The application of WGS in this study also facilitated the construction of a high-resolution phylogenetic framework that further validated the origin of the outbreak. Phylogenetic analysis placed the outbreak cluster strains in close association with isolates from Switzerland, Kenya, Vietnam, and Qatar on a single evolutionary branch, with no distinct long branches or splits. This close genetic relationship suggested that the outbreak strains might share a common ancestor with these international isolates, having subsequently diverged and adapted to the local environmental conditions in China. Such findings highlighted the global interconnectedness of bacterial pathogens and emphasized the importance of genomic surveillance in understanding their evolutionary dynamics and transmission patterns.

This study highlighted several epidemiological characteristics of EAEC, including the occurrence of significant school-based outbreaks in Shandong Province in recent years and the distinctive biochemical traits of ST394 strains and EAEC outbreak isolates. As a genetically heterogeneous and emerging foodborne enteropathogen, EAEC has been associated with both sporadic cases and outbreaks in various countries, such as Japan, South Korea, and Germany, imposing a substantial disease burden on affected populations (Xiong et al., 2025). Despite its global significance, investigations into EAEC-induced diarrheal outbreaks in China remain limited.

EAEC is characterized by extensive diversity in serotypes, VFs, and genetic elements, which are particularly valuable in outbreak investigations. Detecting identical EAEC serotypes or virulence genes during an outbreak is commonly interpreted as evidence of exposure to a common infection source (Suwanawat et al., 2024). In this study, the *app* gene, encoding the dispersin protein, and the *astA* gene, encoding the enteroaggregative heat-stable toxin, were consistently identified among the EAEC strains isolated during the outbreak, with both genes located on the IncFII plasmid. The *app* gene, a non-exclusive and highly prevalent genetic marker among EAEC strains, facilitates bacterial dispersal and colonization of new intestinal regions. Studies have demonstrated that strains harboring the *app* gene exhibit enhanced pathogenicity, correlating with cases of diarrheal diseases, particularly acute episodes in children (Petro et al., 2020; Saha et al., 2024). Meanwhile, the *astA* gene, widely regarded as a hallmark of EAEC strains, is frequently used as a diagnostic marker (Ikumapayi et al., 2017; Ochieng et al., 2023; Kindie et al., 2024). The EAST1 toxin encoded by the *astA* gene promotes chloride secretion, a process closely associated with secretory diarrhea. However, the pathogenicity of EAEC is often attributed to the synergistic or cumulative effects of multiple VFs and toxins, rather than the action of a single toxin (Ikumapayi et al., 2017; Kindie et al., 2024; Saka et al., 2019).

To further investigate the adhesion patterns and pathogenicity of the outbreak isolates, cell adhesion assays were conducted, considering both genetic relatedness and source differences. The results revealed that the outbreak cluster isolates predominantly exhibited the AA pattern, whereas the most distantly related strains displayed the LA pattern. This observation suggested that while the *astA* gene does not inherently confer the AA phenotype, the AA pattern is predominant among EAEC strains (Mujahid et al., 2024). Interestingly, during cell adhesion assays, HEp-2 cells did not display significant morphological changes, such as rounding, cytoplasmic swelling, detachment, or nuclear abnormalities. These findings indicated that the outbreak isolates exhibited minimal cytotoxicity, consistent with the absence of moderate to severe clinical phenotypes among patients during this outbreak. Such observations aligned with the hypothesis that the clinical manifestations of EAEC were generally mild and result from a combination of multiple virulence mechanisms rather than overt cytotoxicity (Kindie et al., 2024; Bahgat et al., 2024).

In recent years, the emergence and spread of MDR and XDR diarrheagenic *E. coli* have posed significant challenges to clinical treatment and public health management (Mujahid et al., 2024; Bahgat et al., 2024; James et al., 2025). This issue is particularly acute in economically underdeveloped regions, where limited alternative interventions and inadequate healthcare infrastructure exacerbate the problem of antibiotic resistance (Hwang et al., 2025). Currently, severe or persistent diarrhea is commonly treated with ampicillin, trimethoprim-sulfamethoxazole, and quinolones (Khafaja et al., 2024). However, due to the toxic effects of quinolones on skeletal development, their use is contraindicated in pediatric populations (Sivarajan et al., 2025). Alarmingly, in this study, isolates resistant to nalidixic acid were identified, with a resistance rate as high as 93.33% (14/15). The irrational use of antibiotics in children raises serious concerns, as it may have long-term adverse effects on growth, development, and immune system functionality (Hwang et al., 2025; Sivarajan et al., 2025; Efrati Epchtien et al., 2025). Consequently, the treatment of diarrhea caused by EAEC in pediatric patients should prioritize safer antibiotic options or supportive therapies, such as rehydration, which can foster the recovery of a healthy gut microbiome (Efrati Epchtien et al., 2025; Akwongo et al., 2025).

Notably, this investigation identified an XDR-EAEC strain from fly specimens collected in a cafeteria, carrying both *mcr-1* and *bla*_CTX-M-132_ resistance genes. The *bla*_CTX-M_ gene family is recognized as the most prominent group of ESBLs worldwide and serves as a paradigm of antibiotic resistance evolution (Lu et al., 2020). These genes are typically located on highly mobile plasmids and are often associated with ISs such as *ISEcp1*, which facilitate their mobilization and horizontal transfer (Zhao et al., 2024). Moreover, *bla*_CTX-M_ frequently coexists with other resistance genes on the same plasmid, amplifying the risk of disseminating multidrug resistance (Efrati Epchtien et al., 2025; Lu et al., 2020; Wang et al., 2024). Colistin, a critical last-line treatment for MDR bacterial infections, is increasingly undermined by the emergence of resistance genes such as *mcr-1* (Sun et al., 2023). The co-occurrence of *mcr-1* and *bla*_CTX-M-132_ on plasmids, as observed in this study, is particularly alarming, as it exemplifies the growing threat of antibiotic resistance convergence. The proliferation of ESBL-producing strains has already necessitated a re-evaluation of colistin as an effective therapeutic option (Lu et al., 2020; Zhao et al., 2024; Chotinantakul et al., 2022). However, the integration and coexistence of these resistance determinants heighten the likelihood of clinical treatment failure and contribute to the emergence of pan-resistant “superbugs” (Shafiq et al., 2022).

The antibiotic resistance of EAEC is driven by the synergistic effects of multiple genes, with some of these interactions directly regulating resistance mechanisms. This study demonstrates a significant correlation between the *floR* gene and TET resistance, consistent with previous findings that *floR* is frequently co-localized with tetracycline resistance genes within multidrug resistance gene clusters in multidrug-resistant strains, leads to a strong phenotypic association between these genes (Trigg et al., 2025). Furthermore, the observed correlation between the *sitABCD* operon and MEM resistance may reflect an indirect regulatory mechanism. Although *sitABCD* itself encodes an iron uptake system and is not directly involved in antibiotic resistance, iron homeostasis plays a global regulatory role in bacterial metabolism. Studies indicate that under iron-limiting conditions, bacteria may upregulate the expression of *β*-lactamases to enhance the hydrolytic activity against carbapenem antibiotics (Chen et al., 2025). *sitABCD* could modulate intracellular iron concentrations to indirectly influence the activity of these enzymes.

Although this XDR strain was isolated from flies, the “One Health” framework underscores the interconnectedness of human, animal, and environmental health in shared ecosystems (Chotinantakul et al., 2022; Shafiq et al., 2022; Macori et al., 2021). The potential transmission of plasmid-mediated *mcr-1* and *bla*_CTX-M-132_ genes from flies to humans highlights the importance of dynamic surveillance of antibiotic-resistant bacteria in animals and the environment (Moser et al., 2021). Proactive monitoring and control measures are imperative to mitigate the risks posed by such resistance genes, thereby safeguarding public health and preserving the efficacy of last-line antibiotics (GBD 2016 Diarrhoeal Disease Collaborators, 2018).

## Conclusion

This study identified the consumption of sauced beef contaminated by a canteen employee as the source of the EAEC outbreak through a combination of epidemiological investigations and comprehensive genomic analyses. Through the integration of phylogenetic analysis, virulence and drug resistance gene and phenotypic detection, it was found that the ST394 type EAEC isolate showed low cytotoxicity characteristics, but the resistance rate of naphthidine acid in adolescents was high, which should be worthy of vigilance. In addition, our investigation detected pan-drug-resistant EAEC strains carrying *mcr-1* and *bla*_CTX-M-132_ resistance genes in fly samples collected from the canteen environment. This finding reveals the potential transmission risk of antimicrobial resistance genes at the human-food-environment interface. To mitigate this, a dynamic pathogen surveillance system for canteens should be established, coupled with strengthened health monitoring of food handlers and rigorous controls throughout food processing. For multidrug-resistant strains, an enhanced AMR surveillance network and tailored prevention protocols must be implemented to disrupt the transmission chains of foodborne pathogens.

## Data Availability

The original contributions presented in the study are publicly available. This data can be found here: http://www.ncbi.nlm.nih.gov/bioproject/1207748/, PRJNA1207748.

## Footnotes

^1^ https://pubmlst.org/
