# Supplementary material for: Enteroaggregative Escherichia coli foodborne outbreak in Shandong Province, China (2023): comprehensive epidemiology and genomic resistance profiling
Source: Front Microbiol. 2025 Jun 27;16:1577277. doi: 10.3389/fmicb.2025.1577277 (PMC12245808; doi:10.3389/fmicb.2025.1577277)

Table S1 Bioinformatics software version and related parameters

Bioinformatics Tool	Verson	Parameters
FastQC	0.12.1	fastqc input.fastq.gz -o ./qc_results
spades	3.15.2	/software/SPAdes-3.15.2-Linux/bin/spades.py -1 -2 -t 24 -k 33,45,55 --isolate -o
prokka	1.14.6	/software/prokka/bin/prokka --outdir --prefix --centre X --compliant
quast	5.0.2	python /software/quast-5.0.2/quast.py -o -t 36
Trimmomatic	0.39	java -jar trimmomatic-0.39.jar PE input_forward.fq.gz input_reverse.fq.gz output_forward_paired.fq.gz output_forward_unpaired.fq.gz output_reverse_paired.fq.gz output_reverse_unpaired.fq.gz ILLUMINACLIP:TruSeq3-PE.fa:2:30:10:2:True LEADING:3 TRAILING:3 MINLEN:36

Table S2 Overview of virulence gene carrying status of EAEC isolated strains

Strain	Virulence gene
BZ37	*kpsD, kpsT, kpsM, gspM, gspL, gspK, gspJ, gspI, gspH, gspG, gspF, gspE, gspD, gspC, csgB, csgG, espL1, espY4, entB, entE, entC, fepB, entS, fepD, fepG, fepC, entF, fes, fepA, entD, rcsB, espY2, espY1, espX1, chuS, chuA, shuT, chuW, shuX, chuY, chuU, shuV, espX5, fimH, fimG, fimF, fimD, fimC, fimI, fimA, fimE, fimB, allB, espR4, espY3, acrB, acrA, gtrA, yagW/ecpD, yagX/ecpC, yagY/ecpB, yagZ/ecpA, ykgK/ecpR, fdeC, gnd, espX4, espL4, aslA, espR1, ompA, aap/aspU, astA*
BZ31	*gspC, gspD, gspE, gspF, gspG, gspH, gspI, gspJ, gspK, gspL, gspM, kpsM, kpsT, kpsD, ompA, csgG, csgB, espL1, espY4, entB, entE, entC, fepB, entS, fepD, fepG, fepC, entF, fes, fepA, entD, rcsB, espX1, espY1, espY2, shuV, chuU, chuY, shuX, chuW, shuT, chuA, chuS, espX5, fimH, fimG, fimF, fimD, fimC, fimI, fimA, fimE, fimB, allB, espR4, acrA, acrB, espY3, gtrA, gnd, yagW/ecpD, yagX/ecpC, yagY/ecpB, yagZ/ecpA, ykgK/ecpR, fdeC, espL4, espX4, aslA, espR1, aap/aspU, astA*
BZ28	*aap/aspU, acrA, acrB, allB, aslA, astA, chuA, chuS, chuU, chuW, chuY, csgB, csgG, entB, entC, entD, entE, entF, entS, espL1, espL4, espR1, espR4, espX1, espX4, espX5, espY1, espY2, espY3, espY4, fdeC, fepA, fepB, fepC, fepD, fepG, fes, fimA, fimB, fimC, fimD, fimE, fimF, fimG, fimH, fimI, gnd, gspC, gspD, gspE, gspF, gspG, gspH, gspI, gspJ, gspK, gspL, gspM, gtrA, kpsD, kpsM, kpsT, ompA, rcsB, shuT, shuV, shuX, yagW/ecpD, yagX/ecpC, yagY/ecpB, yagZ/ecpA, ykgK/ecpR*
BZ27	*aap/aspU, acrA, acrB, allB, aslA, astA, chuA, chuS, chuU, chuW, chuY, csgB, csgG, entB, entC, entD, entE, entF, entS, espL1, espL4, espR1, espR4, espX1, espX4, espX5, espY1, espY2, espY3, espY4, fdeC, fepA, fepB, fepC, fepD, fepG, fes, fimA, fimB, fimC, fimD, fimE, fimF, fimG, fimH, fimI, gnd, gspC, gspD, gspE, gspF, gspG, gspH, gspI, gspJ, gspK, gspL, gspM, gtrA, kpsD, kpsM, kpsT, ompA, rcsB, shuT, shuV, shuX, yagW/ecpD, yagX/ecpC, yagY/ecpB, yagZ/ecpA, ykgK/ecpR*
BZ26	*aap/aspU, acrA, acrB, allB, aslA, astA, chuA, chuS, chuU, chuW, chuY, csgB, csgG, entB, entC, entD, entE, entF, entS, espL1, espL4, espR1, espR4, espX1, espX4, espX5, espY1, espY2, espY3, espY4, fdeC, fepA, fepB, fepC, fepD, fepG, fes, fimA, fimB, fimC, fimD, fimE, fimF, fimG, fimH, fimI, gnd, gspC, gspD, gspE, gspF, gspG, gspH, gspI, gspJ, gspK, gspL, gspM, gtrA, kpsD, kpsM, kpsT, ompA, rcsB, shuT, shuV, shuX, yagW/ecpD, yagX/ecpC, yagY/ecpB, yagZ/ecpA, ykgK/ecpR*
BZ24	*aap/aspU, acrA, acrB, allB, aslA, astA, chuA, chuS, chuU, chuW, chuY, csgB, csgG, entB, entC, entD, entE, entF, entS, espL1, espL4, espR1, espR4, espX1, espX4, espX5, espY1, espY2, espY3, espY4, fdeC, fepA, fepB, fepC, fepD, fepG, fes, fimA, fimB, fimC, fimD, fimE, fimF, fimG, fimH, fimI, gnd, gspC, gspD, gspE, gspF, gspG, gspH, gspI, gspJ, gspK, gspL, gspM, gtrA, kpsD, kpsM, kpsT, ompA, rcsB, shuT, shuV, shuX, yagW/ecpD, yagX/ecpC, yagY/ecpB, yagZ/ecpA, ykgK/ecpR*
BZ21	*shuV, chuU, chuY, shuX, chuW, shuT, chuA, chuS, gspC, gspD, gspE, gspF, gspG, gspH, gspI, gspJ, gspK, gspL, gspM, kpsM, kpsT, kpsD, gtrA, rcsB, gnd, espR4, espL1, espR1, csgB, csgG, ompA, entB, entE, entC, fepB, entS, fepD, fepG, fepC, entF, fes, fepA, entD, allB, acrA, acrB, espY3, fdeC, ykgK/ecpR, yagZ/ecpA, yagY/ecpB, yagX/ecpC, yagW/ecpD, espY2, espY1, espX1, fimH, fimG, fimF, fimD, fimC, fimI, fimA, fimE, fimB, espX5, espX4, espL4, aslA, espY4, astA, aap/aspU*
BZ12	*aap/aspU, acrA, acrB, allB, aslA, astA, chuA, chuS, chuU, chuW, chuY, csgB, csgG, entB, entC, entD, entE, entF, entS, espL1, espL4, espR1, espR4, espX1, espX4, espX5, espY1, espY2, espY3, espY4, fdeC, fepA, fepB, fepC, fepD, fepG, fes, fimA, fimB, fimC, fimD, fimE, fimF, fimG, fimH, fimI, gnd, gspC, gspD, gspE, gspF, gspG, gspH, gspI, gspJ, gspK, gspL, gspM, gtrA, kpsD, kpsM, kpsT, ompA, rcsB, shuT, shuV, shuX, yagW/ecpD, yagX/ecpC, yagY/ecpB, yagZ/ecpA, ykgK/ecpR*
BZ18	*aap/aspU, acrA, acrB, allB, aslA, astA, chuA, chuS, chuU, chuW, chuY, csgB, csgG, entB, entC, entD, entE, entF, entS, espL1, espL4, espR1, espR4, espX1, espX4, espX5, espY1, espY2, espY3, espY4, fdeC, fepA, fepB, fepC, fepD, fepG, fes, fimA, fimB, fimC, fimD, fimE, fimF, fimG, fimH, fimI, gnd, gspC, gspD, gspE, gspF, gspG, gspH, gspI, gspJ, gspK, gspL, gspM, gtrA, kpsD, kpsM, kpsT, ompA, rcsB, shuT, shuV, shuX, yagW/ecpD, yagX/ecpC, yagY/ecpB, yagZ/ecpA, ykgK/ecpR*
BZ16	*acrA, acrB, allB, astA, csgB, entB, entC, entD, entE, entF, entS, espL1, espR1, espX1, espX4, espX5, fdeC, fepA, fepB, fepC, fepD, fepG, fes, fimA, fimB, fimC, fimD, fimE, fimF, fimG, fimH, fimI, galF, gnd, gspC, gspD, gspE, gspF, gspG, gspH, gspI, gspJ, gspK, gspL, gspM, ompA, rcsB, yagW/ecpD, yagX/ecpC, yagY/ecpB, yagZ/ecpA, ykgK/ecpR*
BZ03	*acrA, acrB, allB, aslA, astA, csgB, entB, entC, entD, entE, entF, entS, espL1, espL4, espX1, espX4, espX5, espY1, fepA, fepB, fepC, fepD, fepG, fes, fimA, fimB, fimC, fimD, fimE, fimF, fimG, fimH, fimI, gnd, gspC, gspD, gspE, gspF, gspG, gspH, gspI, gspJ, gspK, gspL, gspM, ompA, rcsB*
BZ05	*acrA, acrB, astA, csgB, entB, entC, entD, entE, entF, entS, espL1, espL4, espR1, espR4, espX1, espX4, espX5, fdeC, fepA, fepB, fepC, fepD, fepG, fes, fimA, fimB, fimC, fimD, fimE, fimF, fimG, fimH, fimI, gnd, gspC, gspD, gspE, gspF, gspG, gspH, gspI, gspJ, gspK, gspL, gspM, ompA, rcsB, yagW/ecpD, yagX/ecpC, yagY/ecpB, yagZ/ecpA, ykgK/ecpR*
BZ01	*acrB, allB, aslA, astA, csgB, entB, entC, entD, entE, entF, entS, espL1, espR1, espX1, espX4, espX5, espY1, fdeC, fepA, fepB, fepC, fepD, fepG, fes, fimA, fimB, fimC, fimE, fimF, fimG, fimH, fimI, gnd, iucA, iucB, iucC, iucD, iutA, ompA, rcsB, yagW/ecpD, yagX/ecpC, yagY/ecpB, yagZ/ecpA, ykgK/ecpR*
BZ32	*acrA, acrB, allB, aslA, astA, csgB, entB, entC, entD, entE, entF, entS, espL1, espX4, espX5, fepA, fepB, fepC, fepD, fepG, fes, fimA, fimB, fimC, fimD, fimE, fimF, fimG, fimH, fimI, fyuA, gspG, gspH, gspI, gspJ, gspK, gspL, gspM, irp1, irp2, ompA, rcsB, ybtA, ybtE, ybtP, ybtQ, ybtS, ybtT, ybtU, ybtX*
BZ15	*acrB, allB, aslA, astA, chuA, chuS, chuT, chuU, chuV, chuW, chuX, chuY, clbA, clbB, clbC, clbD, clbE, clbF, clbG, clbH, clbI, clbJ, clbK, clbL, clbM, clbN, clbO, clbP, clbQ, clbS, cnf1, csgB, entB, entC, entD, entE, entF, entS, fdeC, fepA, fepB, fepC, fepD, fepG, fes, fimA, fimB, fimC, fimD, fimE, fimF, fimG, fimH, fimI, focA, focF, focG, focH, focI, fyuA, galF, gnd, gspC, gspD, gspE, gspF, gspG, gspH, gspI, gspJ, gspK, gspL, gspM, hlyA, hlyB, hlyC, hlyD, iroB, iroC, iroD, iroE, iroN, irp1, irp2, kpsD, kpsM, ompA, papB, papC, papD, papF, papH, papI, papJ, papK, pic, rcsB, sfaB, sfaE, sfaF, sfaY, tcpC, vat, yagW/ecpD, yagX/ecpC, yagY/ecpB, yagZ/ecpA, ybtA, ybtE, ybtP, ybtQ, ybtS, ybtT, ybtU, ybtX, ykgK/ecpR*

Table S3 Setting of the filtering threshold

Parameters	Threshold
QUAL	≥30
DP	10–100X
QD	≥2.0
FS	≤60
MQ	≥50

Figure S1 Comparison chart of alleles

```
                     *        20         *        40         *        60         *        80         *       100         *
BZ21-astA : ATGCCATCAACACAGTATATCCGGAGGCCCGCATCCAGTTATGCATCGTGCATATGGCGCGCAACAGCCTGCGCTTCATGTCATGGAAGGACTACAAAGCAGTCACTCGCGACCTGA : 117
BZ15-astA : ATGCCATCAACACAGTATATCCGGAGGCCAGCATCCAGTTATGCATCGTGCATATGGTGCGCAACAGCCTGCGCTTCGTGTCATGGAAGGACTACAAAGCCGTCACTCGCGACCTGA : 117
BZ24-astA : ATGCCATCAACACAGTATATCCGGAGGCCCGCATCCAGTTATGCATCGTGCATATGGCGCGCAACAGCCTGCGCTTCATGTCATGGAAGGACTACAAAGCAGTCACTCGCGACCTGA : 117
BZ12-astA : ATGCCATCAACACAGTATATCCGGAGGCCCGCATCCAGTTATGCATCGTGCATATGGCGCGCAACAGCCTGCGCTTCATGTCATGGAAGGACTACAAAGCAGTCACTCGCGACCTGA : 117
            ATGCCATCAACACAGTATATCCGGAGGCCcGCATCCAGTTATGCATCGTGCATATGGcGCGCAACAGCCTGCGCTTCaTGTCATGGAAGGACTACAAAGCaGTCACTCGCGACCTGA
```

Figure S2: Correlation analysis between resistance genes and resistance phenotypes

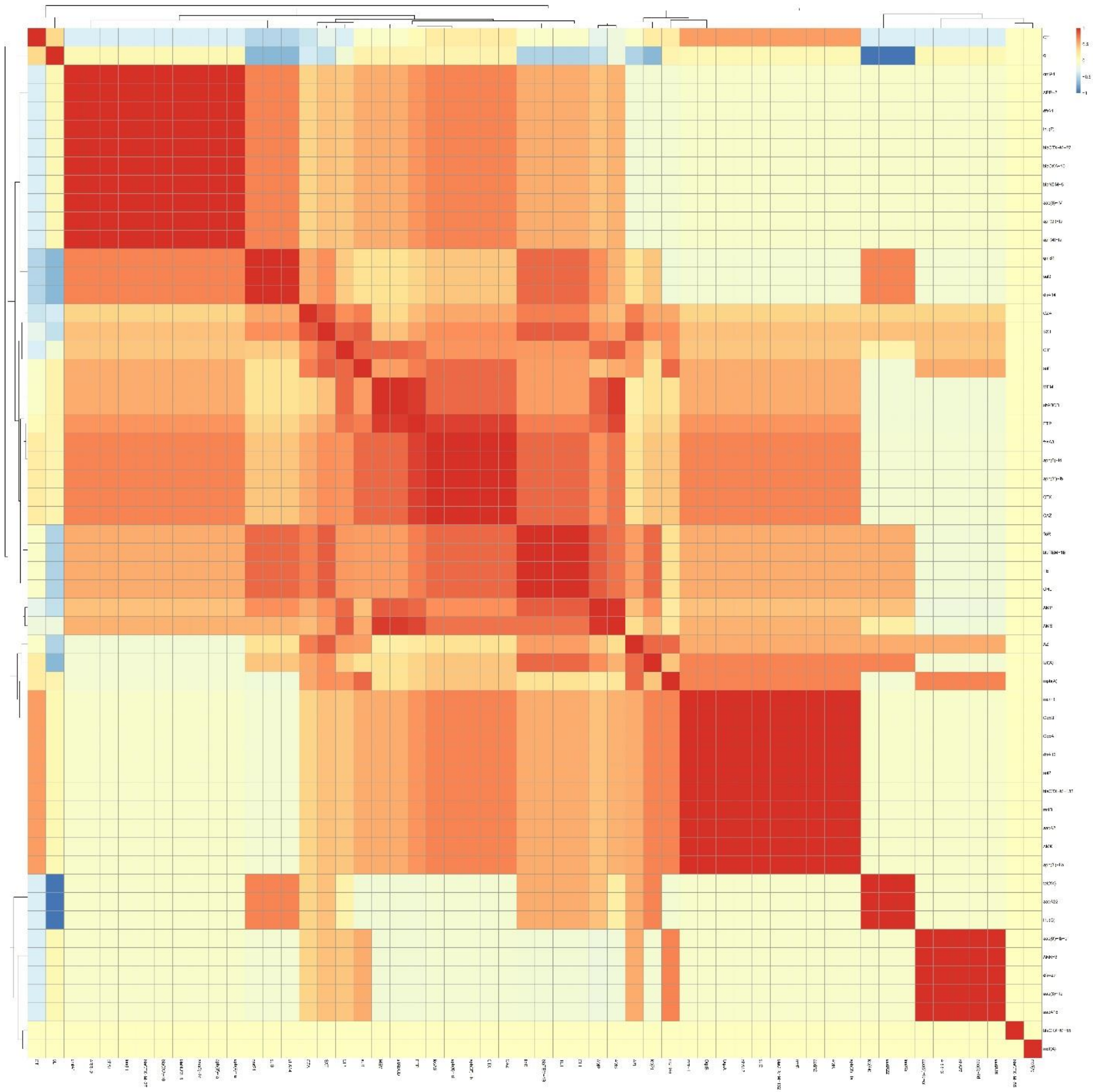

Supplement: Supplementary file 1 [file Data_Sheet_1.pdf]
